# Secure and Provenance Enhanced Internet of Health Things Framework: A Blockchain Managed Federated Learning Approach

**DOI:** 10.1109/ACCESS.2020.3037474

**Published:** 2020-11-11

**Authors:** Mohamed Abdur Rahman, M. Shamim Hossain, Mohammad Saiful Islam, Nabil A. Alrajeh, Ghulam Muhammad

**Affiliations:** 1 Department of Cyber Security and Forensic ComputingCollege of Computing and Cyber SciencesUniversity of Prince MugrinMadinah41499Saudi Arabia; 2 Department of Software EngineeringCollege of Computer and Information SciencesKing Saud University 37850 Riyadh11543Saudi Arabia; 3 Department of Computing, GoldsmithUniversity of London 3163 LondonSE14 6NWU.K.; 4 Department of Biomedical EngineeringCollege of Applied Medical SciencesKing Saud University 37850 Riyadh11543Saudi Arabia; 5 Department of Computer EngineeringCollege of Computer and Information SciencesKing Saud University 37850 Riyadh11543Saudi Arabia

**Keywords:** Blockchain, Internet of Health Things, homomorphic encryption, federated learning, provenance

## Abstract

Recent advancements in the Internet of Health Things (IoHT) have ushered in the wide adoption of IoT devices in our daily health management. For IoHT data to be acceptable by stakeholders, applications that incorporate the IoHT must have a provision for data provenance, in addition to the accuracy, security, integrity, and quality of data. To protect the privacy and security of IoHT data, federated learning (FL) and differential privacy (DP) have been proposed, where private IoHT data can be trained at the owner’s premises. Recent advancements in hardware GPUs even allow the FL process within smartphone or edge devices having the IoHT attached to their edge nodes. Although some of the privacy concerns of IoHT data are addressed by FL, fully decentralized FL is still a challenge due to the lack of training capability at all federated nodes, the scarcity of high-quality training datasets, the provenance of training data, and the authentication required for each FL node. In this paper, we present a lightweight hybrid FL framework in which blockchain smart contracts manage the edge training plan, trust management, and authentication of participating federated nodes, the distribution of global or locally trained models, the reputation of edge nodes and their uploaded datasets or models. The framework also supports the full encryption of a dataset, the model training, and the inferencing process. Each federated edge node performs additive encryption, while the blockchain uses multiplicative encryption to aggregate the updated model parameters. To support the full privacy and anonymization of the IoHT data, the framework supports lightweight DP. This framework was tested with several deep learning applications designed for clinical trials with COVID-19 patients. We present here the detailed design, implementation, and test results, which demonstrate strong potential for wider adoption of IoHT-based health management in a secure way.

## I. Introduction

With the availability of the Internet of Health Things (IoHT), more health data is becoming available for the healthcare industry to benefit from [1], [2]. With the recent advancements in deep learning applications, IoHT data can even be automatically parsed with high accuracy, and people’s health conditions can be monitored without human intervention. Hospitals can deploy advanced deep learning applications to perform triage and diagnosis, thereby reducing the wait time to see doctors. However, the IoHT collects very sensitive health and ambience data that requires privacy protection and security guarantees [3]. Many governments around the globe have imposed restrictions on centralized data collection processes. Hence, traditional deep learning applications that rely on a central, powerful cloud machine that accumulates a vast amount of IoHT data, and on training a model accurately are encountering regulatory restrictions [4]. In addition to regulatory restrictions, cloud-based central machine learning applications might not be suitable for training a massive amount of IoHT data [5]. To address the security and privacy issues of IoHT data, current advancements in edge learning allow private health data be trained locally at hospital premises [6]. State-of-the-art machine learning algorithms, e.g., federated learning (FL), allow distributed and collaborative training, where the training data does not need to be shared with a cloud entity. This allows the private data to remain under the owner’s control. Some salient features of FL are 1) the possibility of parallel training, 2) edge and local training with full autonomy, 3) a reduced burden on data management, 4) minimum data labeling requirements, and 5) FL can provide personalized learning through transfer learning [7]. In a typical IoHT-based health application, onboard IoHT sensors perform the sensing, an FL algorithm performs the learning and reasoning, and decision-making is done by either humans or an AI algorithm based on data analytics. Traditional FL poses several challenges for IoHT-based applications. Some notable features of FL are 1) FL may have a very large number of diversified types of clients with various capacities and edge resources, 2) each FL node may own different quantities and qualities of non-independent, identically distributed (non-IID) and privately owned training data, 3) there may be delays and unstable communication overheads in aggregating the models, and 4) there may be security and privacy concerns related to malicious nodes or an aggregating server [8], [9]. The training model is vulnerable to cyber-attacks, such as generative adversarial networks. The authors in [10] showed that vanilla FL architecture may expose some individual private data of the edge node to a malicious cloud model aggregation node through the analysis of shared gradients. Hence, the owner of the IoHT data, the hospital authority that co-owns the electronic health and medical records, and the governments that provide citizen’s healthcare services need security and privacy guarantees for IoHT data [11].

Privacy and data provenance in the IoHT for deep learning applications can be realized in several ways [12], [13]. Researchers have tried to propose techniques to ensure that privacy is not compromised during the collaborative training process. The authors in [15] developed a private data leakage method in an FL environment. A quantitative trustworthiness metric was defined in [16], where the authors measured IoHT data provenance by introducing privacy and obfuscation. For example, compared with a single training node, the accuracy of the training data can be increased by adding more federated nodes, where some of the nodes have superior quality and a higher number of datasets. The privacy of each local federated node can be ensured through local training at the edge. Although FL can provide a certain degree of privacy over private data, if the partial or full dataset needs to be shared with another entity when the training needs to be anonymized or confidential, differential privacy (DP) or homomorphic encryption (HE) can be implemented [17]. In another recent study [21], a weighted FL was proposed that used multi-party computation (MPC) protocol addition and multiplication over encrypted space. For example, additive HE can be applied to mask model parameters during local gradient updates on a batch of gradients [22]. Moreover, a model that is trained either on a central node or at edge/federated nodes can be encrypted as well [23]. Trained models that are shared between different entities within the distributed nodes can be de-identified. Because the FL model allows more than one node to take part in the training process, malicious nodes taking part in and compromising either the training, model aggregation, or inferencing processes should be identified, and appropriate secure defense mechanisms must be applied as a control mechanism.

One of the flawed assumptions of vanilla FL is that the central aggregation server is trustworthy. To overcome this, researchers have proposed the usage of a fully decentralized gradient aggregation process [24]. Blockchain has strong cryptographic strength and a decentralized approach to performing secure transactions among trustless entities [25], [26]. To provide IoHT data provenance, blockchain and off-chain have shown great promise. Off-chain, such as the InterPlanetary File System (IPFS), has been popular for storing training datasets in a decentralized fashion, and blockchain has gained popularity for managing the trust and provenance of trustworthy federated nodes, their datasets, the reputation of each node, the accuracy of the models each node generates, the immutability of the global model, etc., [27]. The authors in [28] proposed a provenance management scheme for the Ethereum-based blockchain. Some researchers have also proposed other aspects of security, e.g., the work presented in [17] proposed the trusted execution environment (TEE), in which a hardware-based enclave takes care of attesting the confidentiality, integrity, privacy, security, and authentication of both data and the parties that are involved in the secure computation. The FL environment may involve nodes having software/hardware-based implementation, such as Intel’s Software Guard Extensions (SGX), and ARM’s TrustZone. Another interesting lightweight security and provenance protocol was introduced in [29], where the wireless link strength of IoHT nodes was used as a measure. In another dimension of provenance, the authors in [30] studied the decision provenance of deep learning application as a pipeline to check the trustworthiness of AI algorithms. Both blockchain and machine learning were surveyed in the context of IoHT data security and privacy by the author in [31].

Applying a deep learning process [8] to healthcare [14], [18], [19], [83] and IoHT [48] data at the edge has been a subject of focus in recent studies [32]. For example, in the age of tremendous scrutiny of health data privacy and stringent security requirements, researchers have resorted to deep learning models that allow secure training and model aggregation and dissemination of either the model or the inferencing results. FL allows multiple private nodes containing private IoHT data to use a secure deep learning model and to retrain on local data to produce a custom model. Later, either centralized or decentralized aggregation of all related local models can take place, based on certain criteria such as the reputation of the locally trained model, the training time, and the quality and accuracy of the trained model, etc. The researchers in [33] demonstrated such a decentralized FL in the context of designing a COVID-19-based collaborative radiometric diagnosis tool. Other researchers have tried to improve the efficiency of decentralized FL by introducing federated transfer learning [34]. For example, the researchers in [35] proposed a model by which each participating federated node could maximize its gain, maintain the privacy and security of the local data, and lower communication costs while the overall system ensured the security of the global model. In another dimension, the researchers in [36] tried to address the issue of diversity of types of federated nodes, their security and privacy requirements, and their performance and resource constraints, and proposed a deep reinforcement learning model that could learn such requirements and provide an optimal solution. Some researchers have categorized the privacy and security of the IoHT in terms of training the FL model based on independent and identically distributed (IID) and non-IID health datasets [23].

To support the advancements of the IoHT in a secure and privacy-protected manner, it is required that the deep learning model and the training dataset be within the private network of a user [37]. However, due to the complex nature of the deep learning application life cycle, e.g., the high-quality training and testing of an IoHT model from a rich training and validation dataset, obtaining an appropriate model is generally out of reach for the data owner. A data owner can be any person having the IoHT in his/her possession. While the privacy and security of personal and private data is of utmost concern, the model for training this private data requires either an externally trained model to be available to the private data on the edge, or the private data needs to be uploaded to the owner of the model. In either situation, security and/or privacy comes into play. If the model is encrypted, it can be shared with the private data owners to benefit from the trained model without the need to share private data with the model owner. On the other hand, if the private data is first encrypted and then shared with the model owner in such a way that the model owner can work on the encrypted dataset and return an encrypted inferencing result, then the model owner is not able to access either the private training dataset or the inferencing result. For example, researchers at Microsoft have introduced CRYPTFLOW, which can convert a regular TensorFlow model to MPC cryptographic protocol, and allow multiple federated nodes to apply a publicly known deep learning algorithm to their private datasets [38]. While secure MPC is still vulnerable to an inferencing attack, some researchers have proposed a hybrid architecture in which secure MPC and DP can be applied for better accuracy and data quality [18].

Thanks to the recent advancements in deep learning for encrypted computing, encryption schemes such as full or partial HE and secure MPC are gaining popularity. This has led to deep learning framework and library designers such as Google and Facebook offering frameworks such as TensorFlow Encrypted, Syft Keras, and PyTorch Opacus. These models allow both the private data owner and the external model owner to encrypt their data and model respectively and perform secure training and model inferencing in a distributed environment, without needing to trust any particular entity. Another approach suggested by researchers in [39] uses differentially private generative adversarial network (GAN) to generate secret tokens for detecting malicious attackers, and differentially private stochastic gradient descent to handle privacy leakage. Because FL requires distributed federated nodes to communicate in a secure and privacy-oriented way, researchers have proposed a compression technique for efficient communication, and additive HE and DP for data and model security and privacy [40]. Before the start of the FL aggregation process, non-benign training results can be filtered out, and only those malicious edge nodes can be blacklisted [41]. In another study [4], a two-phase FL process was proposed in which Phase 1 allowed voting for some trusted committee member from the federation, and in Phase 2 the actual FL takes place under the guardianship and privacy-protecting watchful eye of the committee members.

The recent COVID-19 pandemic [41], [47], [48] has presented the possibility of using the IoHT on a massive scale. Due to the human-to-human transmissibility of the pathogen, IoHT-based healthcare is becoming commonplace. The existing secure and privacy-oriented FL techniques show promising prospects. However, few researches have used the above advancements as a proof of concept for managing COVID-19. In this paper, we present the following novel contributions:

• In order for IoHT stakeholders and federated nodes to bring trust to the provenance of training data and shared models, we leverage blockchain and off-chain so that the provenance data itself is protected from tampering and unauthorized access. Blockchain replaces the untrustworthy central gradient aggregator with a trustworthy, tamper-proof gradient mining and decentralized consensus-based aggregator.
• To add an extra layer of security at the blockchain nodes that are responsible for aggregating the gradients, we propose using the Intel SGX TEE, which will use a secure enclave for the local model aggregation process. At the end of aggregation within the secure enclave, the encrypted global model’s hash is stored in the blockchain for further sharing.
• To lower the communication and computational burden on the FL edge nodes, HE of MPC is proposed, which combines the secret sharing and privacy of the model gradients. To lower the probability of model inferencing attack that exposes individual training data, we propose a certain degree of DP to balance between the privacy and model accuracy. Secure aggregation of models allows us to prevent a poisoning attack, in which a malicious FL node might introduce a backdoor poisonous model that could add bias to the training data and tilt or “poison” the inference.
• We developed a provenance collection and management graph that leverages blockchain and off-chain to track the lineage of the data, model, and transaction history of the deep learning process. Authenticated and authorized clients may query the history and genuineness of the deep learning process, dataset used, and training process in a secure way. The module was trained with a supervised learning process to recognize malicious intruder nodes by maintaining reputation scores.
• We designed several COVID-19 applications that use deep learning to classify IoHT data. The sensory data generated by IoHT are passed through a series comprising a provenance module, DP module, and confidential module before they become part of the deep learning ecosystem.
• We propose a secure transfer learning model in which a global COVID-19 diagnosis-related model trained through secure FL can be downloaded from a blockchain address, and an edge node can build a personalized local model without compromising privacy or security.
• We tested the security and provenance methods on 23 COVID-19-related deep learning applications in which a variety of datasets, models, diversified types of edge federated nodes, and performance metrics were used.

The rest of this paper is organized in the following way. Section II presents a literature review, while Section III illustrates a detailed system design, followed by the implementation details in Section IV and the test results in Section V. Finally, we provide our conclusions in Section VI.

## II. Literature Review

### A. Differential Privacy

Blockchain has been used in the context of DP. Because artificial noise is being added to prevent a privacy leak through data query, the authors in [42] proposed blockchain to track the total and individual privacy budgets, how much additional noise was added to unique queries, and how much more noise could be allowed before degrading the quality of dataset and privacy cost. In another effort [43], researchers proposed collaborative game-based training data sharing, and made the updated model available on the blockchain. An appropriate incentive for a genuine training data provider is woven into the framework. To prevent a malicious attack and reduce the amount of consensus computing on the blockchain network, the authors in [44] designed a blockchain-based FL network to store the global model and the incremental updates. DP was used in combination with FL and the blockchain for IoT data used in smart home monitoring [45].

FL has been used by researchers to provide DP. An interesting work in [46] benchmarked the usage of FL in IoHT edge nodes. For example, the work presented in [47] uses one-shot FL that tries to find a privacy balance between two extreme phenomena, in which fully private nodes suffer from a very low amount of data, while the fully central aggregation of data models is done on a central node. The privacy of IoT data for unmanned aerial vehicles was studied in the context of FL in [48]. FL suitable for IoHT-based edge computing was illustrated in [49]. The security and privacy aspects of IoHT data through distributed FL in the context of a 6G network were studied in [51] and [52]. In a similar study, the security and privacy of AI training or inferencing data and models within 6G networks were surveyed in [51] and [52]. The authors in [53] proposed a self-healing FL network, which could collaboratively train and detect anomalous nodes. To address privacy, poisoning attacks, and latency issues, the work presented in [54] used blockchain where miners approved the uploaded models from federated edge nodes through a consensus mechanism. To avoid data privacy leaks within 5G ultra-dense mobile edge networks, the authors in [55] proposed FL administered by blockchain. To provide auditing and accountability in terms of the contribution of collaborative FL processes, the authors in [56] designed BlockFlow, which incentivizes genuine contributors while isolating malicious contributors. To ensure the anonymity and privacy of IoT data in edge devices, the authors of [57] proposed a blockchain based on a decentralized FL architecture. The security and privacy of IoHT data used within the data aggregation cycle of FL has been studied by the authors of [58] in a smart home context.

### B. Secure Deep Learning Models

The security and privacy of deep learning applications over 5G edge networks were surveyed in [59]. In order to protect the sensitive data available to network functions or network slices of a 5G network, and protect the confidentiality of local deep learning model updates, the authors in [63] proposed a protocol on top of FL within 5G networks. IoT-based traffic data was subjected to FL by the authors in [61], where an individual train line’s private data was trained using an SVM RBF kernel function, and the global training module was administered by a secure blockchain smart contract. Blockchain is gaining popularity for verifying the integrity and authenticity of on-device FL models [62]. Because 5G allows a very large number of federated clients to join in model creation, the authors in [63] proposed a byzantine-resilient distributed learning suitable for 5G networks. Although applied in the vehicular IoT domain, the work shown in [64] uses a permissioned blockchain and a local directed acyclic graph to secure the deep learning model parameters. The blockchain ensures the reliability of the shared data that are used for the deep reinforced learning process within the FL network. Researchers have proposed a consortium blockchain for finding the reputation of each federated node so that the IoT nodes or mobile federated nodes that have either malicious data or poor-quality data can be filtered [65]. A secure deep learning model called secureSVM was developed by the authors in [66] that did not require a trusted third party. The IoHT data was first encrypted using the Paillier homomorphic cryptosystem, and then the encrypted IoHT data was stored in the blockchain for immutability and provenance. Data poisoning and inferencing attacks on FL algorithms were surveyed in [67]. The survey also proposes the design factors of a resilient FL model.

### C. End-to-End Encrypted Deep Learning Application

Deep learning applications using IoHT require confidentiality and security. To encrypt facial features for emotion recognition [68], an encrypted facial recognition algorithm called Wasserstein generative adversarial network encryption can be used. A demonstration of using full HE called MORE (matrix operation for randomization or encryption) shows [69] that the training can take place on an encrypted dataset, and finally the inferencing algorithm can classify the encrypted X-ray images. The proposed end-to-end encryption algorithm was applied on the MNIST dataset, and the performance was satisfactory compared with plain text deep learning. To support crowdsourcing in blockchain-enabled FL with high security, the authors in [70] proposed the ElGamal public key cryptographic scheme to protect the communication among federated blockchain worker and requester nodes. Blockchain has been used by researchers to monitor and control global model update and aggregation from local federated models [71]. The authors in [22] proposed an HE that could encrypt a batch of local model update gradients to lower communication and computational costs. To protect IoT data privacy, the authors in [72] proposed a secure parameter aggregation technique called a gated recurrent unit, which supports FL without disclosing private IoT data.

### D. Secure IoHT

In the past, researchers proposed lightweight security protocols for the IoHT [73], [74]. For example, XOR, addition, subtraction, and a hash-based authentication protocol were proposed for bringing trust to IoHT device-device authentication [75]. A lightweight secure key exchange algorithm was designed for IoHT-based EHR data exchange scenarios in which a three-way handshake takes place between an IoT device, a gateway, and the IoT cloud [76]. IoHT data privacy and the secure aggregation of FL models were presented in [77], where hardware-based security protection and a Diffie–Hellman key exchange protocol were used to make an Ethereum native encryption toolkit. Bringing trust to IoHT data was the central focus of the research presented in [78]. A privacy-protected IoHT framework was designed in [79], where IoHT data with provenance and audit trails were delivered to authorized subscribers. The authors in [80] designed a cryptosystem that could secure IoHT data during transmission between two endpoints. The cryptosystem was successfully tested with medical image transmission. Federated transfer learning of IoHT data was proposed in [81]. A recent initiative of the Internet Engineering Task Force to manage DDoS or man-in-the-middle attacks on the IoHT suggested using a manufacturer usage description (MUD) as a unique signature [82]. An FL architecture can use the MUD signature with the IoHT to provide security for deep learning applications at the edge.

### E. Provenance Using Blockchain

Blockchain has gained trust in providing provenance, data integrity, authentication, and immutability for the IoHT [84]. A detailed survey on support for IoT application security through blockchain can be found in [85]. The authors in [86] designed a multi-tenant blockchain application that guaranteed scalability, data integrity, and data privacy within a permission blockchain. BlockDeepNet is an architecture that allows secure sharing of private data between collaborative IoT nodes, so that a sufficient amount of IoT is available for the training data required for the deep learning application, while blockchain provides the confidentiality, authentication, and integrity of the collaborative IoT nodes [87]. Blockchain and an IPFS-based off-chain solution were used in to provide secure mobile cloud access [88] and secure EHR data sharing between patients and medical service providers, while keeping the personal data on the mobile device private. The probabilistic method has also been used in combination with blockchain to provide authentication and authorization for IoT data transactions [89]. Blockchain has been used to provide data assurance and resilience in an IoT network [90]. A high-throughput and scalable blockchain data structure based on DAG, which was designed for Industrial IoT, was proposed in [91].

Blockchain and deep learning have been used for provenance in the food industry, where deep learning was used for fruit classifications [92]. The authors in [93] showed that the trust of IoHT data by different parties can be ensured through smart contracts containing trusted IoT zones. In order to resist IoT data tampering, and data provenance and impersonation attack, the work presented in [94] used blockchain smart contracts and physical unclonable functions (PUFs). Blockchain-based smart meter data provenance and a lightweight hash-based security algorithm were proposed in [73] to detect meter data tampering. A software defined network offering blockchain-as-a-service was presented in [95], where two different secure deep learning model training scenarios were presented. The blockchain-empowered training data is first uploaded to a decentralized IPFS file system for data provenance, and then either a partially decentralized or a fully decentralized co-operative model training takes place. Blockchain has been proposed by researchers to thwart data poisoning and membership inferencing attacks by not allowing malicious or unreliable FL participants [96]. Blockchain has been used for failure detection in IoHT devices using FL [97]. Secure and privacy-maintained data and model sharing with the help of a permissioned blockchain was illustrated in [98].

## III. System Design

**FIGURE 1.**
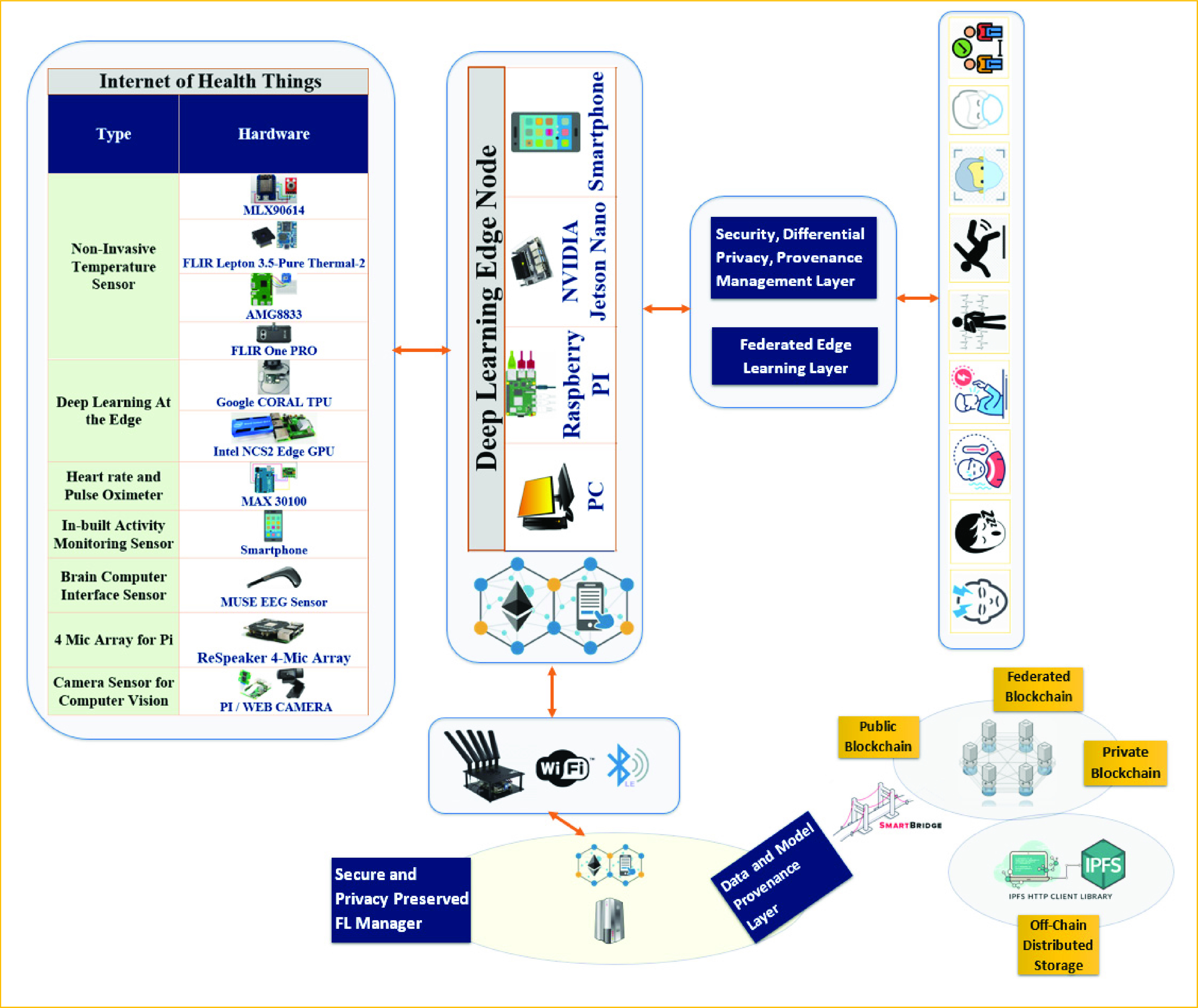
High-level security, privacy, and provenance supporting deep learning applications using Internet of Health Things.

In this section we will elaborate on a provenance collection and management system that leverages blockchain and off-chain to track the lineage of the data, model, and transaction history of the deep learning process. Figure 1 shows a high-level overview of the proposed system. We selected a set of IoHT sensors, as shown in Figure 1, that can help us in managing COVID-19 symptoms and diagnosis, or in pandemic management. The IoHT interfaces with the edge nodes. The edge nodes have a GPU and own local, private data for local training and inferencing. The edge nodes are also capable of acting as a local blockchain client or federated worker and perform DP and homomorphic operations. The edge nodes are capable of inferencing COVID-19-related symptoms. The communication module of the edge nodes first performs DP and then securely shares the encrypted model and/or training data to the blockchain dApp for further processing. The blockchain client processes the block creation and shares the smart contract of the hybrid blockchain node for global analysis. The differentially private IoHT raw data is first stored in the IPFS repository, and the hash of the training data or model location from the IPFS is stored in the blockchain for provenance and collaborative model training. To add another layer of security at the decentralized aggregator nodes, we considered Intel SGX to provide a TEE. This allows us to use the regular cloud (e.g., Microsoft Azure) for hosting the blockchain miner nodes and the smart contracts. The federated nodes share their secure trained model to the enclave of the TEE, which performs model aggregation. This cryptographically protects the model parameters from being hijacked.

Authenticated and authorized clients can query the history and genuineness of the deep learning process, dataset used, and training process in a secure way. The module is trained with a supervised learning process to recognize malicious intruder nodes by maintaining reputation scores. As for FL, we embedded both the federated stochastic gradient descent and federated averaging approach. Although federated averaging introduces a greater burden on edge nodes, it provides better accuracy in the training process. Also, we assumed our edge nodes, such as Raspberry PI, had sufficient memory, and added GPUs such as Intel NCS2 or Google CORAL TPU. We targeted several COVID-19 applications that used deep learning to classify IoHT data. The sensory data generated by the IoHT are passed through a series comprised of provenance module, DP module, and confidential module before they become part of the deep learning ecosystem.

To avoid leaking gradient information to the aggregating or decentralized malicious federated nodes, we used full HE. The additive and multiplicative operations in convolution, max pooling, and other types, except the activation function, can be performed in a homomorphic way, while the activation function is first converted to polynomials. We incorporated a secure transfer learning model in which a global COVID-19 diagnosis-related model trained through secure FL can be downloaded from a blockchain address, and an edge node can build a personalized local model without compromising privacy and security. Finally, we test the security and provenance methods on 23 COVID-19 related deep learning applications, in which a variety of datasets, models, diversified types of edge federated nodes, and performance metrics were used.

As a design metric, we considered both imbalanced and identical, and non-identical data distribution. We also considered the overhead of implementing these security and privacy measures on edge devices such as smartphones and Raspberry Pi. Some of the metrics that we considered were the amount of federated training time, overhead of encrypted communication, on-board edge device power consumption, and amount of edge memory needed. We planned to achieve lower communication overhead even with millions of learning parameters on IoHT devices connected to GPU-enabled edge devices. In the following section, we will show a sample design of a federated deep learning application, which allows provenance, DP of an immunization certificate generation, maintenance, and sharing with trusted stakeholders. The application can be securely deployed to multiple IoHT edge nodes.

### A. Design of High-Level Scenario of Secure and Provenance-Awares IoHT

COVID-19 [13], [99] has disrupted our daily lives to a great extent. Because no vaccine is available yet, every precaution must be taken to avoid getting infected, which requires that social distancing is maintained everywhere, suspected subjects are isolated from other people, and the status of subjects with symptoms and reports of COVID testing are maintained dynamically. Until now, service providers, e.g., shopping malls, airports, schools, hospitals, and restaurants, etc., do not have the ability to know a visitor’s COVID status, which makes the staff and other customers vulnerable. One widely used way to recognize COVID-suspected people, which uses thermal cameras or IR, fails when it comes to asymptomatic COVID patients. Moreover, after testing, sharing the patient’s status between authorities and service providers is not feasible, due to the way health data sharing is not secure. There is a need to share COVID-19 health status that can be stored securely and privately, so that it can be shared when needed to prove health status. Thanks to the recent advancement in Blockchain, IoT, and social media technologies [20], [50], [60], [100], [101], we can develop such a health service [107]. Written transactions on blockchain and off-chain can be intelligently trained by deep learning applications [102]. We used the Paillier algorithm for additive operations, and RSA for multiplicative homomorphic operations and AES-256 symmetric key encryption algorithm in our FL architecture [87].

**FIGURE 2.**
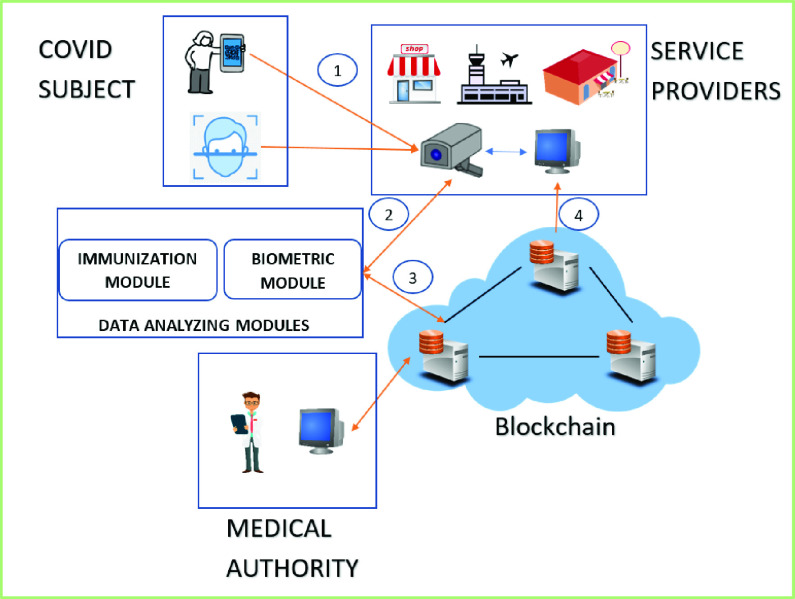
High-level system architecture.

In this study, we propose a comprehensive solution to the above-mentioned problems by developing an enhanced system, as shown in **Figure 2**, which has applications for all three entities concerned, namely the COVID-19 subjects, the health authority, and stakeholders such as government, businesses, and wherever health status needs to be checked for safety. I this scenario, the monitoring authorities authorize access to the sensitive health status of each subject while extending sharing access to the owner of the health status, i.e., the citizens. The authorized health agency generates a QR code-based profile of each visiting subject through the medical authority dApp. The dApp allows uploading one’s basic health data related to the COVID-19 symptoms and facial features as biometric data, which are analyzed by our deep learning algorithm. Transactions are stored on the blockchain, and the encrypted raw data, such as facial features, are stored off-chain. At any given time, the medical authority is able to verify and update the health status of the user. The stakeholders, also called service providers, can check a customer’s health status through the public dApp, which uses a subject’s QR code and face scan data for health status checks in public places. As a proof of concept, we present our developed system with the features mentioned above.

**FIGURE 3.**
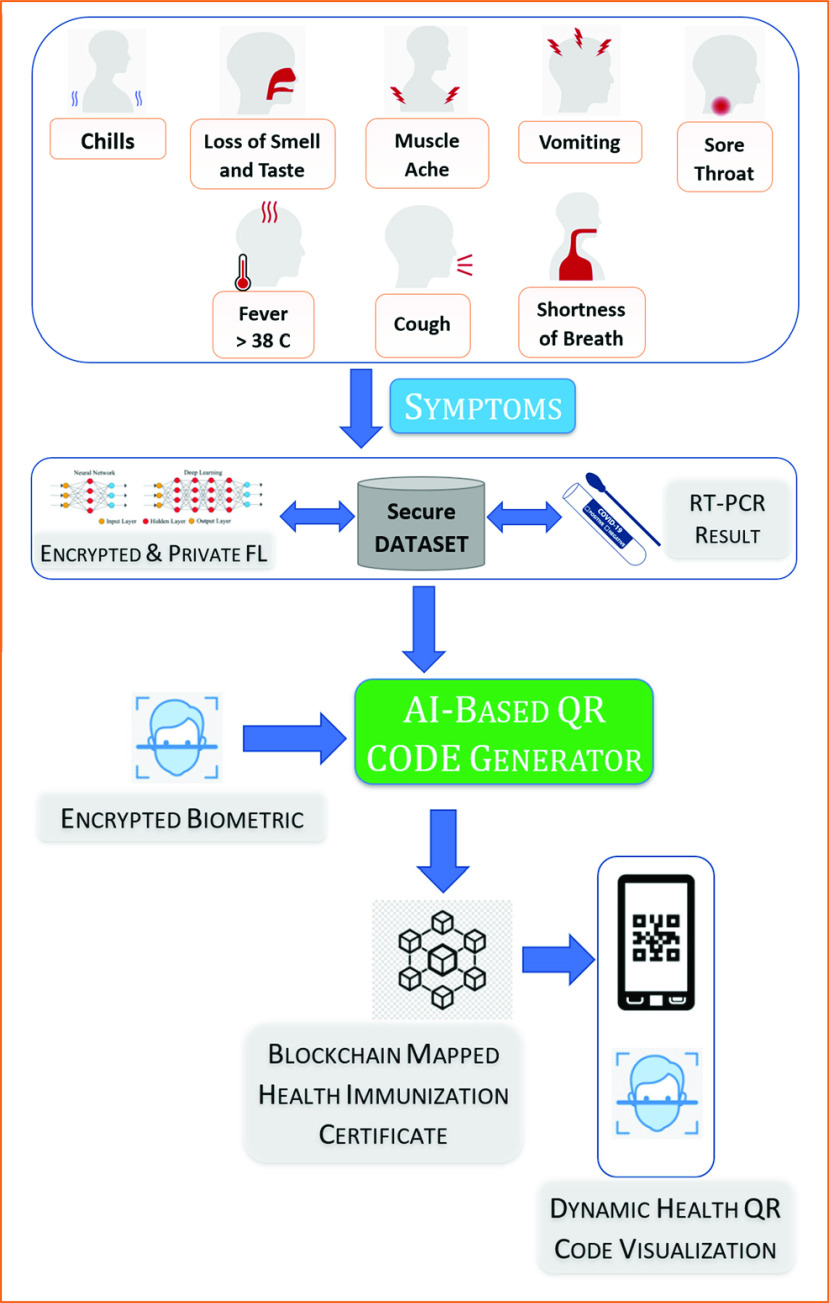
Immunization module for generating QR codes.

The system that we built for demonstration is based on three applications and two integrated data analyzing modules. **Figure 3** shows the high-level architecture of the system. Using the citizen dApp, any subject can create his/her profile, add biometric credentials, and upload the perceived symptom data to the blockchain and off-chain. The authorized medical hospitals can generate health immunization certificates and upload the COVID status of a citizen to the blockchain. **Figure 3** shows the process of generating a health immunization certificate and subsequently visualizing the most recent health status in the form of color-coded QR codes. Finally, the third app is for the public service providers, such as hospitals, malls, schools, and restaurants, etc., that can check the COVID status of every user by 1) scanning their health immunization certificate (QR code), or 2) checking deep learning-based biometric credentials. The steps for the business stakeholder application are shown in **Figure 2**.

**FIGURE 4.**
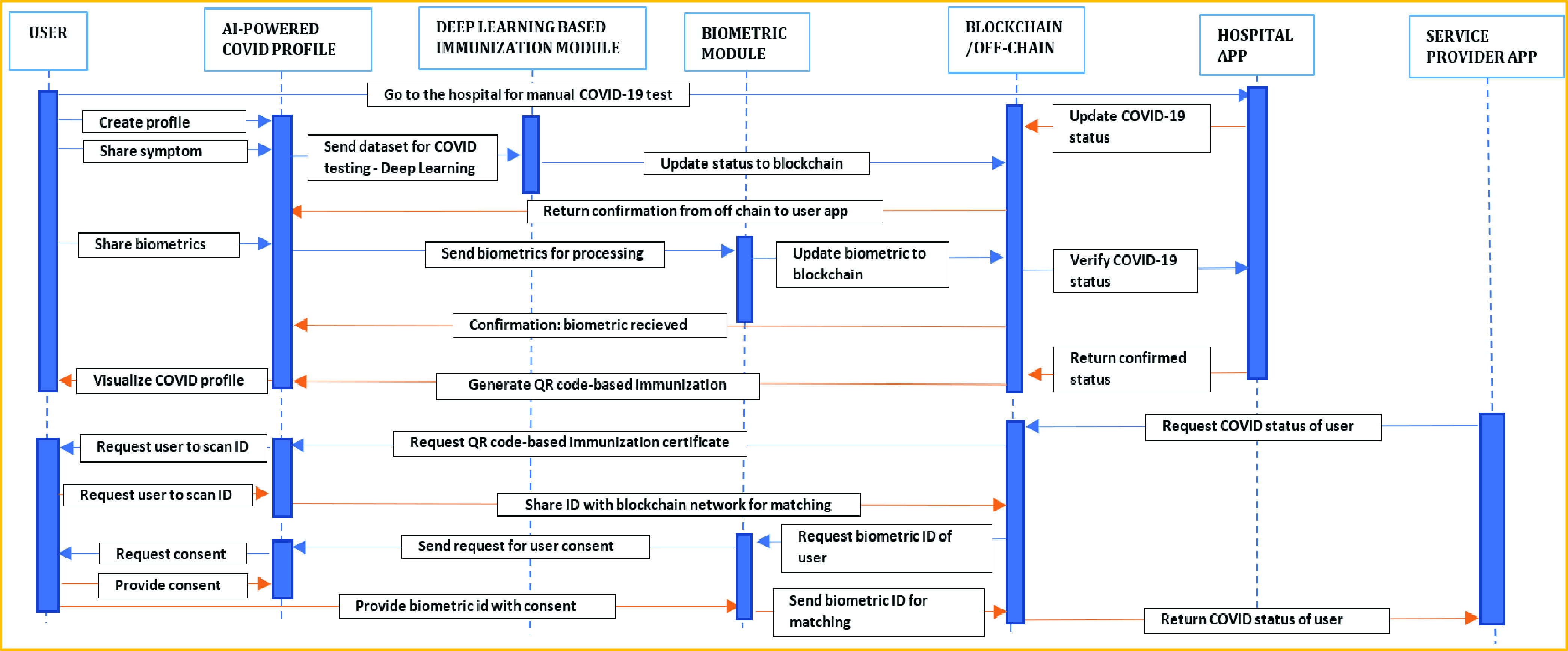
Sequence of secure user interaction and system output.

These sets of applications are powered by two modules. One is the immunization module, which uses deep learning algorithms to detect COVID from symptom data. The other is the biometric module, which uses deep learning (CNN) for face recognition. We used Ethereum and Hyperledger to set up the distributed ledger system, and IPFS for off-chain. **Figure 4** shows the interaction among different entities.

## IV. Implementation

### A. IoHT-Based Secure and Private Deep Learning Applications on the Edge

As part of the proof of concept, we deployed the following applications in our secure cloud.^1^^1^http://advancedmedialab.com/UPM-AI-COVID19/demo-AI.html

• *Contactless biometric recognition-based entrance system:* This application was developed to allow contactless entry to prevent COVID-19 virus transmission. We used a door lock sensor [103], which interfaces with a camera module. Both are integrated with a Raspberry Pi. The Raspberry Pi runs the deep learning application.
• *Handwritten text and medical document processing:* This module uses PyTesseract for the optical character recognition (OCR) process. This module allows a subject to use a smartphone or normal camera to read medical prescriptions or other types of labels.
• AI chatbot for medical systems: An AI chatbot is used to support an ongoing query about COVID-19 and to follow up.
• *AI-based symptom collection:* Different types of questionnaire-based symptom collection with natural language processing are performed by this module.
• *Male and female gender classification from random images:* This application used deep learning to classify subjects as male or female using a regular public camera installed at the entrance of a home.
• *Recycled waste classification:* This application classified waste types using a normal camera stream.
• *Medical report/text recognition:* We have developed a specific deep learning module that can recognize different fields in an electronic health report, either from a PDF soft copy version or from a printed medical report and suggest follow-up intervals.
• *Multiple object detection:* To solve the challenge of multiple object detection from a single camera, we used YoloV5 on an edge device that had a GPU such as Intel NCS or Google CORAL TPU.
• *AI-based visual assistance for blind:* This application was built to support those who have diabetic retinopathy with severe Nonproliferative Diabetic Retinopathy (NPDR) and Macular Edema. The visual assist application uses a smartphone camera running YoLoV5 to detect human faces, persons with/without masks, moving objects, etc., and generate alerts.
• *Face mask detection:* This module was trained to recognize people with/without masks using a public or smartphone camera so that an appropriate alert can be generated.
• *Wet and dry surface detection:* This module allows a public facing camera to detect wet and dry surfaces and generate alerts for fall detection.
• *Food/fruit recognition:* This is a computer vision-based application to classify types of fruits and their quality.
• *Fall detection:* This module detects a fall using YoloV5 and computer vision. An alert is generated after a fall is detected.
• *Emotion analysis:* Different types of facial expression for emotion such as joy, sadness, anger, normal, pain, etc. can be recognized by this module [68].
• *Secure contact tracing:* This module uses blockchain and deep learning applications for contact tracing.
• *Drowsiness/tiredness detection:* Using facial features, this module can detect drowsiness and yawning, and generate alerts.
• *On-premise pill, medicine recognition:* Using this module, pills can be registered and then recognized.
• *Remote surveillance and movement application:* This application allows recognizing the remote movements of objects of interest.
• *Fever Detection using a thermal camera:* Through thermal camera, this module can detect human faces and deduce fever.
• *Social distancing violation alert:* This application monitors social distance violations using computer vision and generates alerts.
• *Covid-19 status tracking using dynamic color-coded QR codes:* Figure 2 explains the detailed design of the deep learning application.
• *Immunity certificate authentication via Ethereum smart contracts:* Figure 3 explains the detailed design of one sample deep learning application.

### B. Training

We used open-source and free datasets for each of the applications mentioned above. For some specialized cases, we used our own datasets, developed for internal usage for this research only. We used three NVIDIA Jetson Nano and 4 Raspberry Pi 4 8 GB federated nodes. Each edge node hosts several IoHT to carry out diversified types of COVID-19-related health services [104]–[106]. The edge nodes had either built-in GPUs, e.g., Jetson Nano, or external GPUs, e.g., Intel NCS2 or Google CORAL TPU. As an edge client, we used smartphones with moderate GPUs, e.g., S20 Ultra 5G and Note 10+ 5G, and a laptop with NVIDIA RTX 2080 and an 8 GB GPU. All the edge nodes were operated as blockchain and deep learning clients. These edge nodes also acted as federated nodes that communicated with an edge full blockchain node for decentralized, differentially private, and homomorphic training and model sharing.

We implemented the training model using Keras with a TensorFlow Encrypted (TFE) backend. The model was trained with and without DP. The training with DP was done so that the model was not exposed to, or could not remember or leak private or personal training data. The TFE supported three types of secure multi-party computing (SMPC) modes, namely Pond, Secure NN, and ABY-3. To aggregate the model gradients from different federated clients, we tested an additive secret sharing cryptographic algorithm that did not rely on a trusted server for aggregation.

We also tested the Opacus DP library while training the PyTorch models. We leveraged autograd hooks while computing batches of per-sample gradients. We configured CUDA 10.2-based crypto-secure tensors running on an NVIDIA RTX 2080Ti GPU in parallel, where the torchcsprng library uses AES-128-bit encryption keys. This allows the framework to provide private predictions, through which any raw training dataset is encrypted end to end. Private predictions can be done using algorithms such as Syft Keras. The Syft Keras ensures that the model is also encrypted, i.e., the weights and the parameters. The Syft Keras sequential class allows for sharing the encrypted model to several federated nodes, applying encrypted computation, and classifying the results on encrypted data. As for the TEE enclave, we used a PySyft implementation of Intel SGX through GRAPHENE SGX.

### C. Setup of the Environment

**FIGURE 5.**
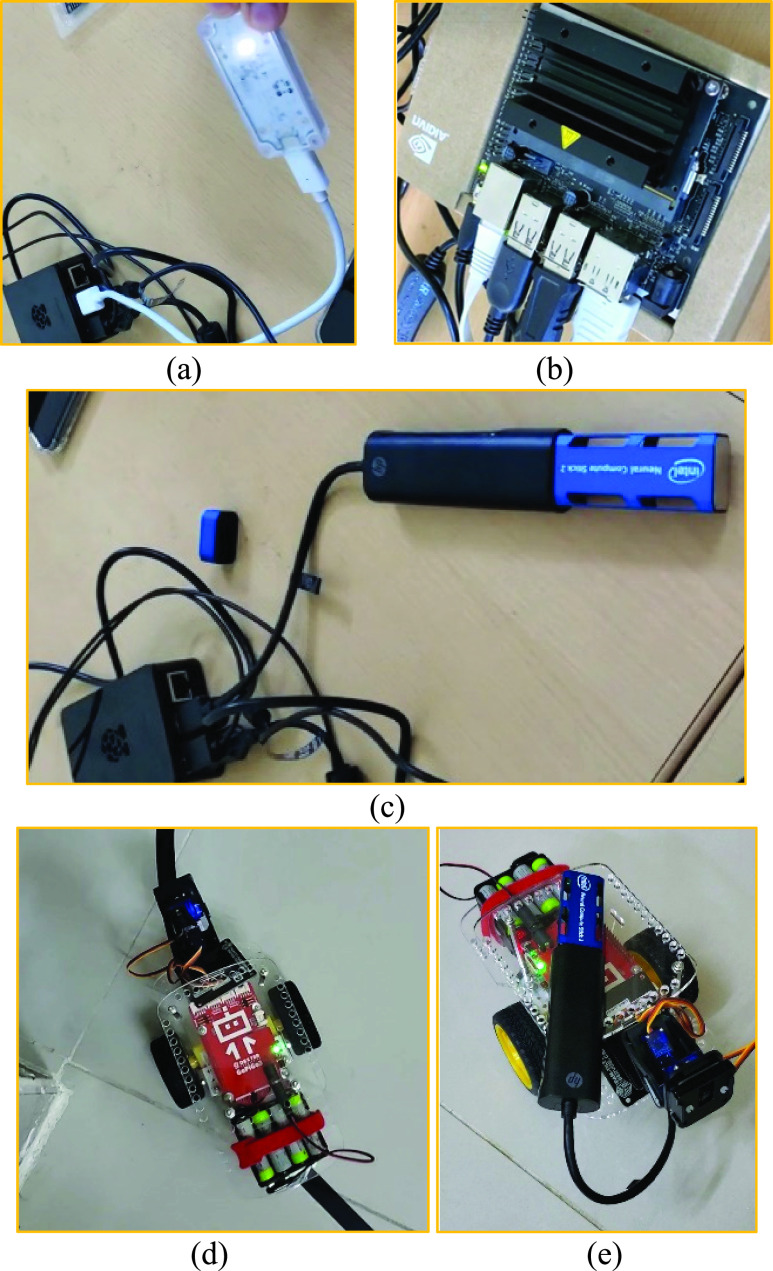
Setup of the edge nodes in different federated learning scenarios: a) raspberry Pi with a Google CORAL TPU GPU, b) NVIDIA Jetson NANO edge device with built-in GPU, c) Raspberry Pi with Intel NCS2 edge GPU, d) a Raspberry Pi with gopigo robotic body equipped with IoHT and computer vision hardware, and e) same as d but with edge GPU.

Figure 5 shows the setup of both static and dynamic edge nodes in a decentralized environment. The edge nodes vary in their GPU, processing capabilities, and types of IoHT sensors, depending on the type of application they serve. The following code snippet shows the configuration of three deployed Raspberry Pi 4 acting as federated nodes running Ubuntu 18.04 and connected to a 5G router.

**FIGURE 6.**
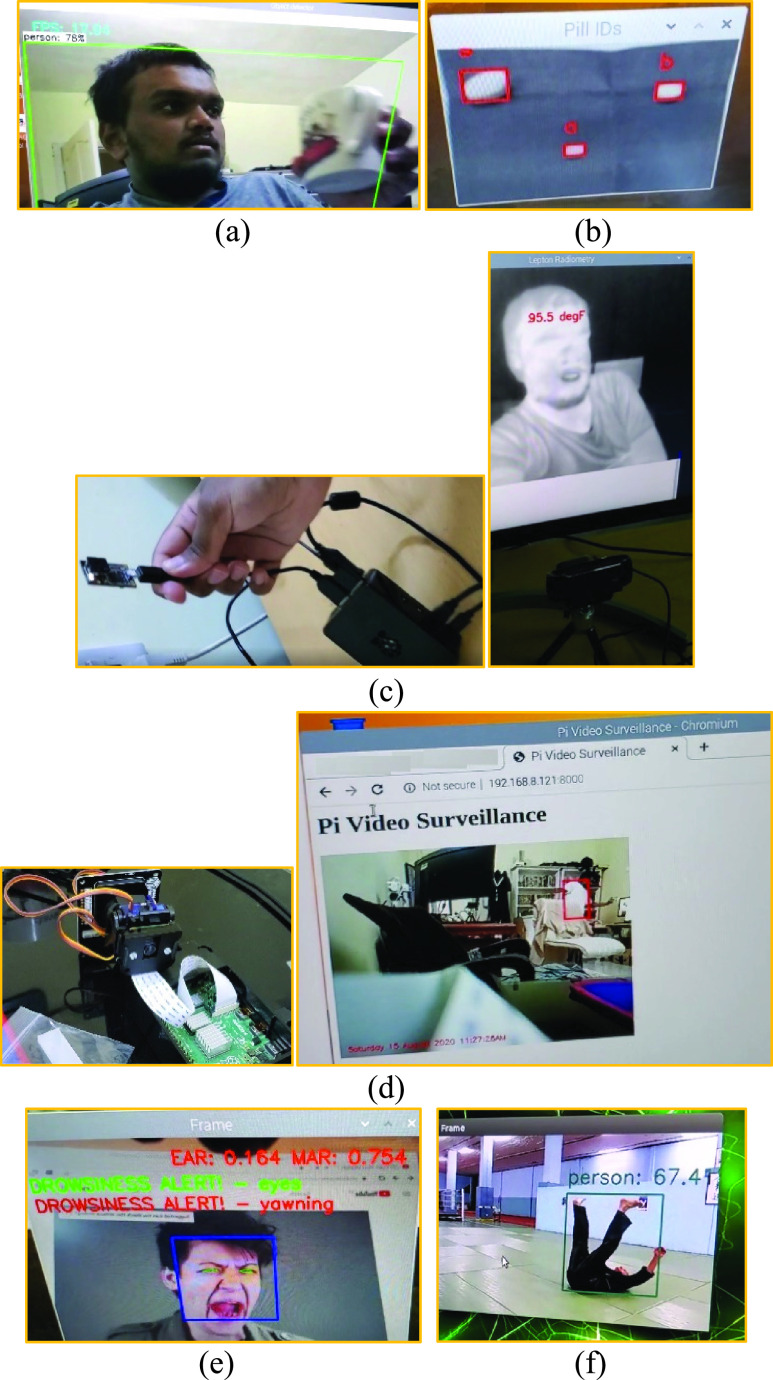
Federated learning applications running at edge nodes (a) Object recognition at a remote edge in terms of frames/second of a Raspberry Pi having an Intel NCS2 (b) Raspberry Pi with an attached Pi camera to recognize pills in a live camera feed (c) Fever detection from a FLIR thermal camera and the corresponding Raspberry Pi host (d) Raspberry Pi assisting in a remote human activity surveillance operation (e) Raspberry Pi attached to a Google Coral TPU physiological state monitoring application, and (f) NVIDIA Jetson NANO edge node tracking, recognizing, and alerting the detection of a human fall.

Figure 6 shows the snapshots that we took from some of the selected deep learning applications. Figure 6 (a) shows the object recognition performance at a remote edge in terms of frames/second of a Raspberry Pi having an Intel NCS2 GPU. Figure 6 (b) shows the interface that allows a Raspberry Pi with an attached Pi camera to recognize pills in a live camera feed. Figure 6 (c) shows fever detection from a FLIR thermal camera and the corresponding Raspberry Pi host of the IoHT nodes. Figure 6 (d) shows a Raspberry Pi assisting in a remote surveillance operation where human activity is being recognized by the Raspberry Pi federated node. Figure 6 (e) shows a Raspberry Pi attached to a Google Coral TPU health monitoring application, which tracks tiredness, weakness, pain, yawning, etc., from a Pi Camera feed. Finally, Figure 6 (f) shows an NVIDIA Jetson NANO edge node tracking, recognizing, and alerting the detection of a human fall. The applications use the HE, DP, and FL configurations as detailed in Section III.

### D. Blockchain-Based Provenance

**FIGURE 7.**
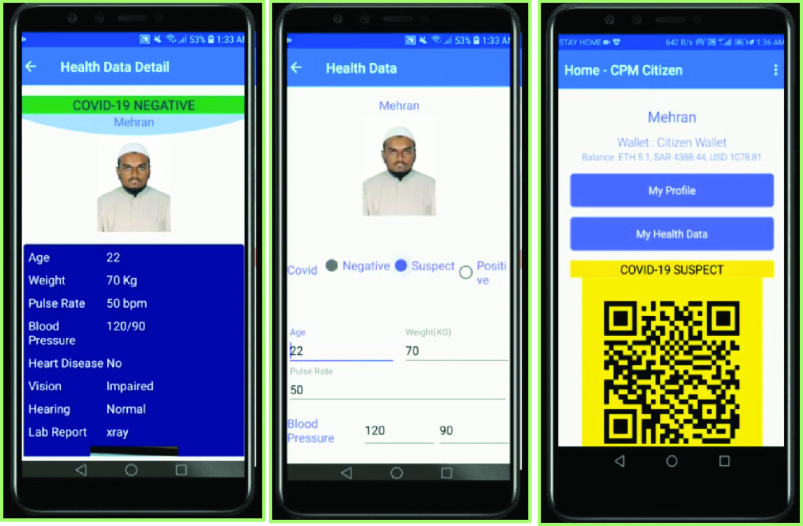
Blockchain provenance: Medical authority updating COVID status that reflects on the user’s application.

**FIGURE 8.**
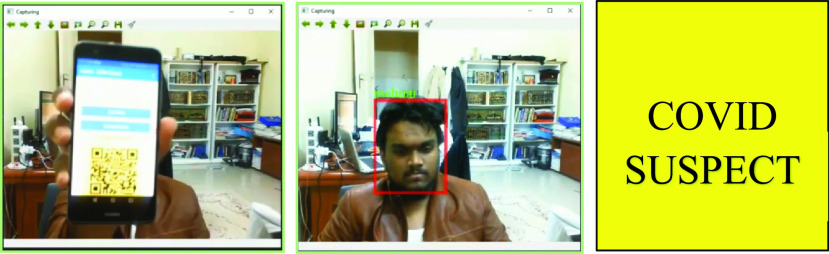
Blockchain provenance: Service providers checking entering person’s COVID status.

We developed a set of dApps for citizens, medical authorities, and service providers, which are shown in Figure 7 and Figure 8. Figure 7 shows the process of medical authorities verifying a user’s COVID status and updating it from negative to suspect, which is updated simultaneously in the user’s app. Figure 8 shows the same person sharing his health immunization certificate and biometric credentials via the service provider’s software. Ethereum-based smart contract were used for IoHT data provenance. The smart contract platform consists of a provenance storage layer, generic provenance layer, and specific provenance layer. For example, we designed 23 deep learning-based applications as a proof of concept, in which each of the applications had certain provenance requirements specific to the application, while there were generic provenance features that all the applications shared.

## V. Test Results

**FIGURE 9.**
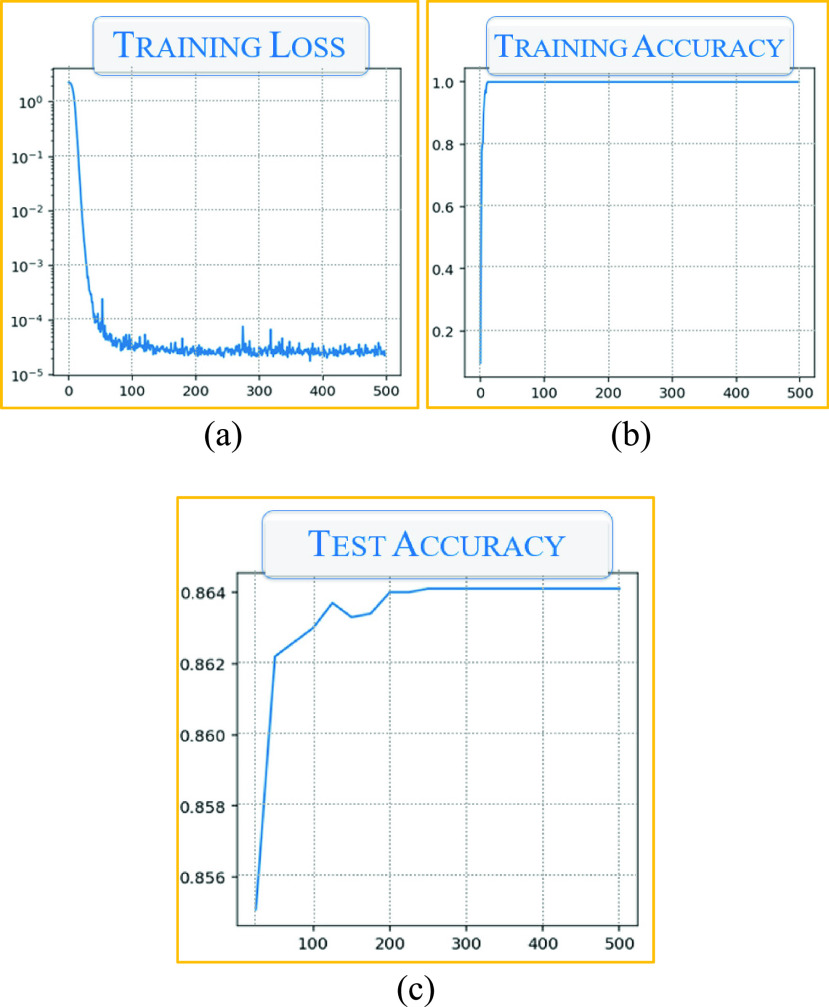
Model training and test metrics of the QR Code Application (a) Training loss (b) Training accuracy, and (c) Test accuracy.

In this section, we discuss the test results. Figure 9 shows the average training loss, training accuracy, and test accuracy of the deep learning application shown in Figure 7 and Figure 8. As can be seen, despite adding DP noise, encryption of the local gradients, and a provenance guarantee, the training loss is within a tolerable limit. As for the accuracy, the application attained above 90% accuracy in training, and above 85% in testing.

**FIGURE 10.**
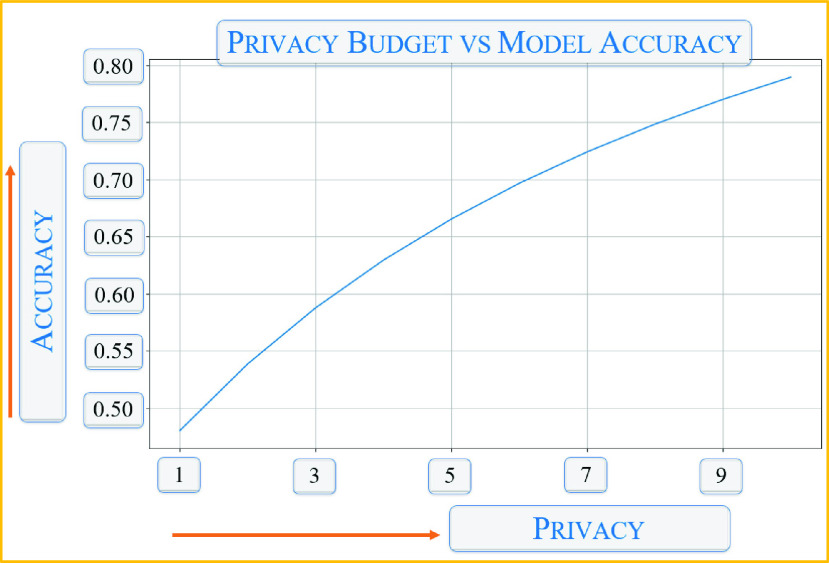
Comparison of privacy budget vs. model accuracy.

Table 1 shows the metrics of the six deep learning applications shown in Figure 6. As can be seen, the average accuracy of all the tested applications are above 89%. This is because the convolution, max pooling, and higher-polynomial approximation of the activation operations in deep learning applications are performed in the cipher domain. Also, the DP requires adding a safe noise threshold, looks for privacy budget limits, and then denoises—all causing compromise in the accuracy and loss metrics. Figure 10 shows the testing result of the privacy vs. accuracy observation and tradeoff. While the privacy budget increases, the test accuracy also increases up to a certain limit.

**TABLE 1:** Auto Grading Metrics

Average Loss	Accuracy (%)	Application
0.00078	91.77	Figure 6 (a)
0.00091	89.25	Figure 6 (b)
0.00145	90.54	Figure 6 (c)
0.00237	92.42	Figure 6 (d)
0.00321	91.64	Figure 6 (e)
0.01025	89.55	Figure 6 (f)

We observed another privacy metric, which was the number of iterations in FL. As can be seen from the above figure, the DP guarantee decreases, while the number of iterations in the communication round in FL increases. This is because each iteration adds a certain amount of noise.

We also tried to capture the energy consumption of the IoHT due to the overhead of the encryption and DP. As can be seen from the dataset in Figure 12 (a-b), we tried to create a random distribution of the sensors in the federated nodes. Figure 12 shows the IoHT that collects vital information in an invasive way, e.g., ECG and EEG sensors, or in a non-invasive way, such as using a thermal camera. In both cases, the energy usage shows that deep learning applications requiring security, provenance, and privacy consume a moderate to high energy level. Figure 13 shows the predicted values vs. the true values projected for the applications shown in Table 1. As can be seen, the overall distribution shows a linear relationship in a broad sense with fewer outliers.

**FIGURE 11.**
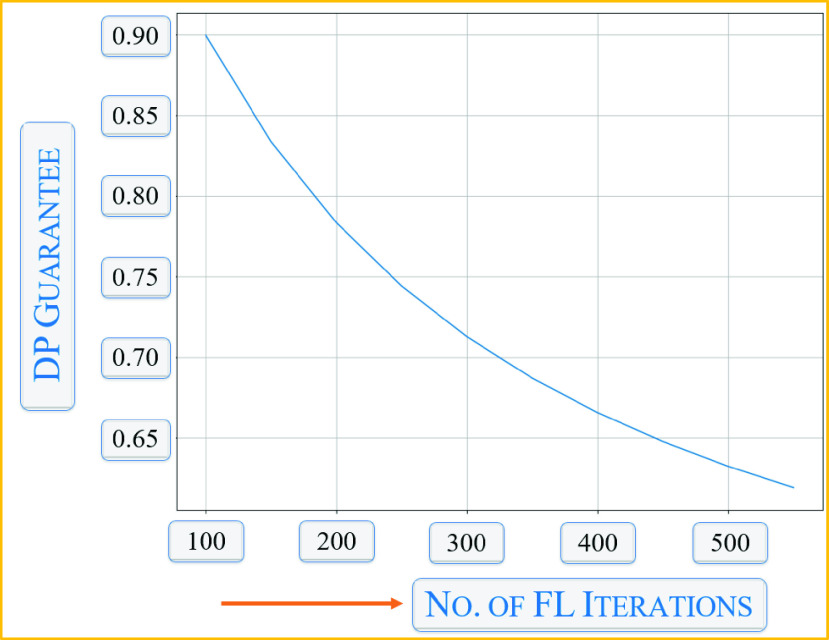
Effects of differential privacy on number of FL rounds.

**FIGURE 12.**
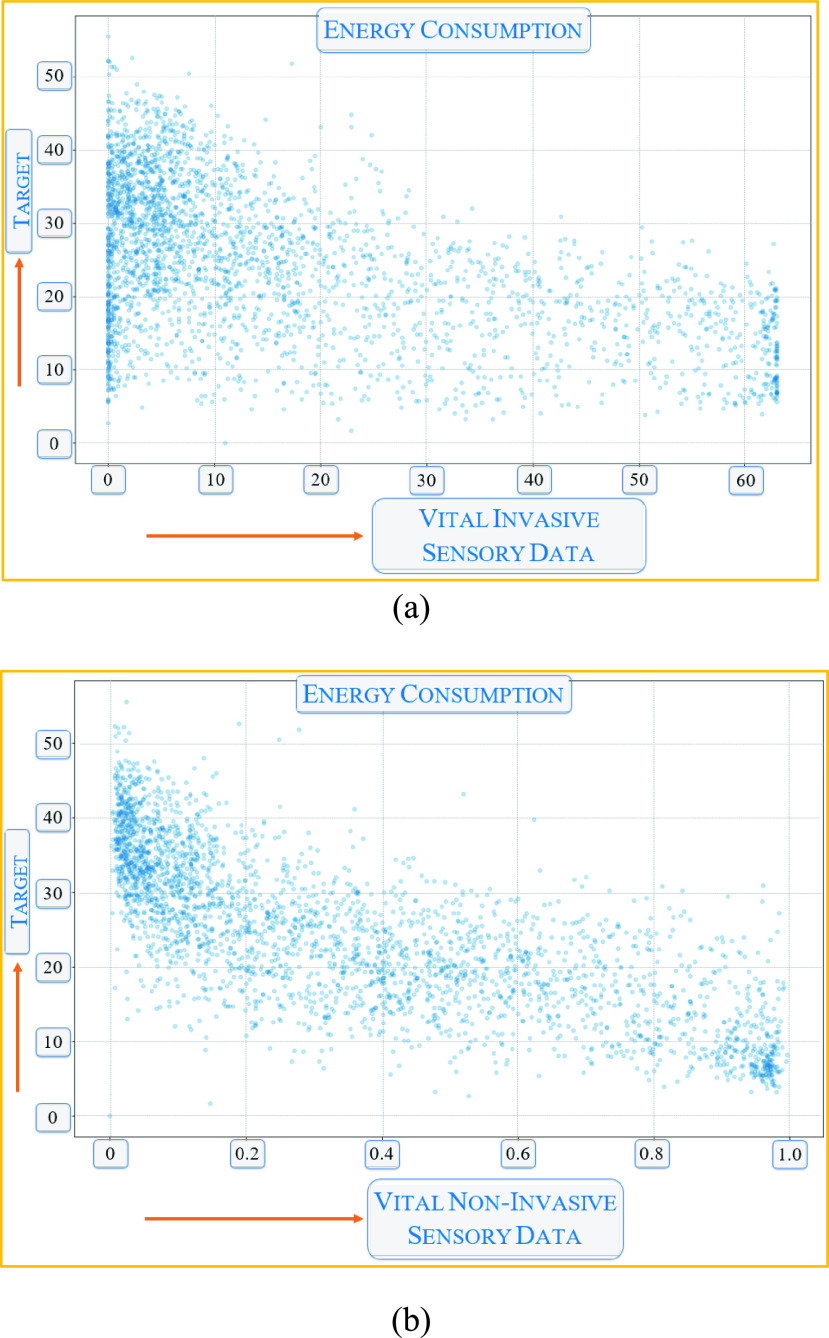
Effects of privacy and provenance on energy consumption in the IoHT (a) Vital Invasive IoHT sensors, and (a) Vital Non-Invasive IoHT sensors.

**FIGURE 13.**
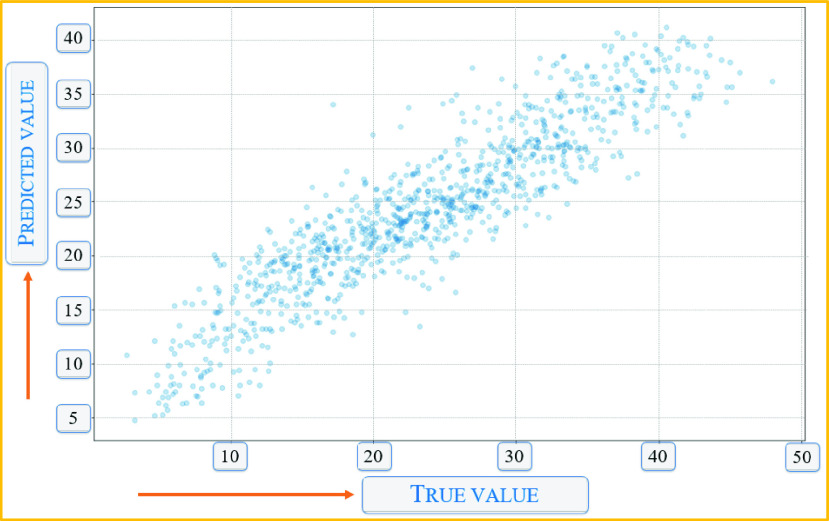
Predicted vs. true value comparison of encrypted and differentially private deep learning applications.

**FIGURE 14.**
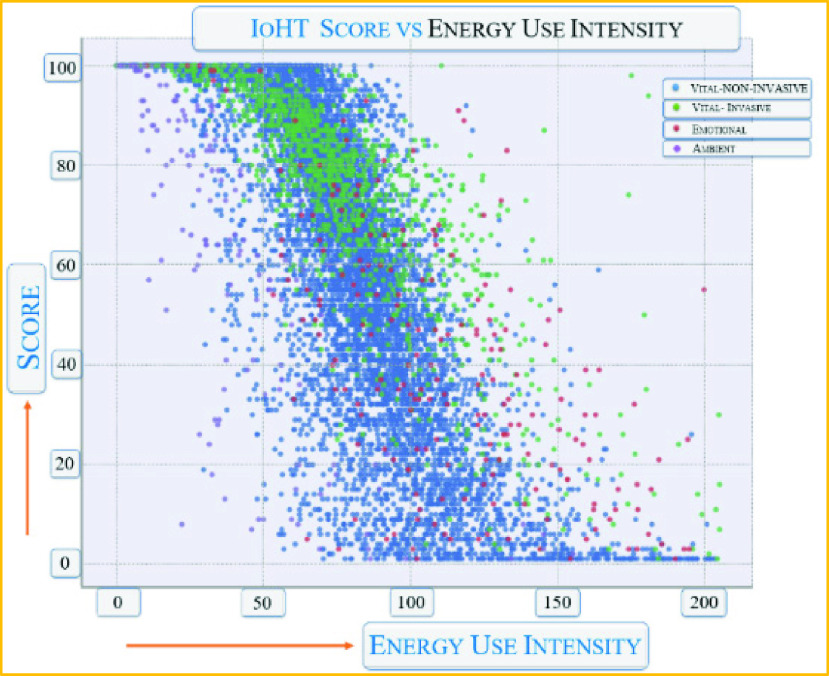
Effect of high-intensity energy consumption due to computing related to IoHT in FL.

Figure 14 shows the pattern and score of IoHT sensors when it comes to energy usage. As the demand for energy increases, the score decreases. This shows the weaknesses of using edge nodes such as Raspberry Pi for full-fledged deep learning computing nodes.

**FIGURE 15.**
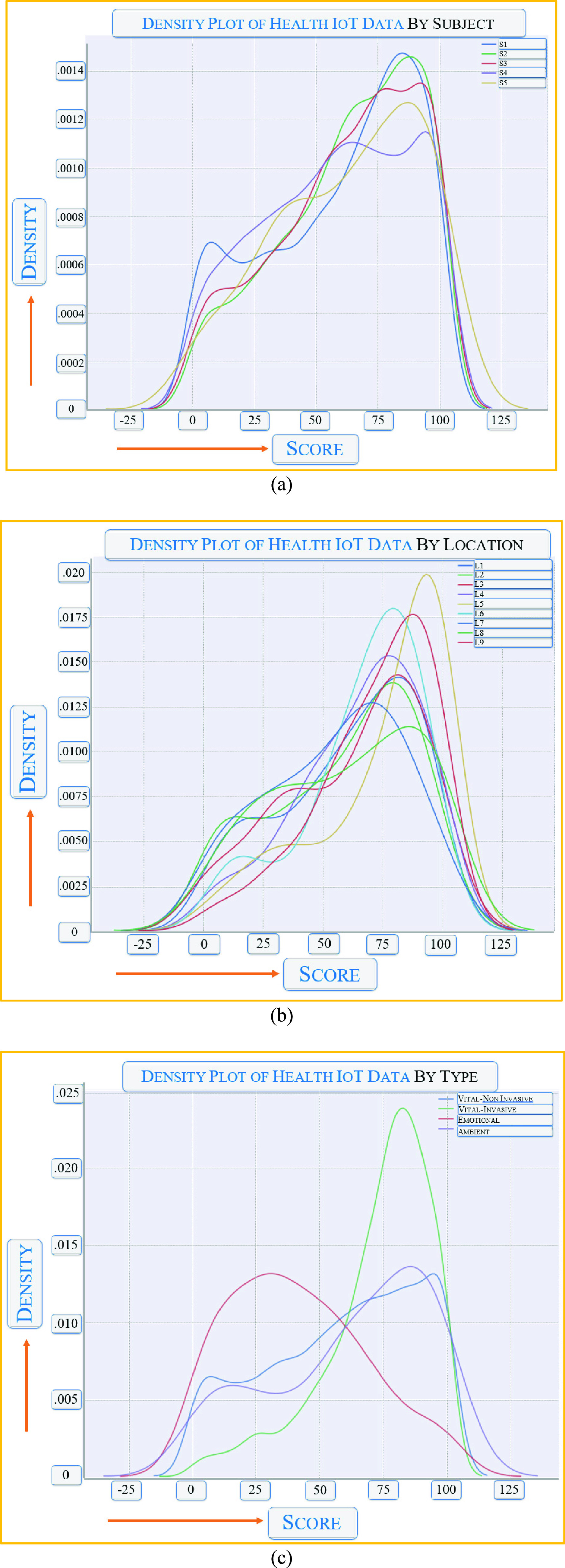
Effect of energy density in the IoHT in FL: (a) observed variations by subject, (b) location, and (c) type of IoHT.

We also tried to see the effect of variation in usage, resource type, available bandwidth or other types of factors affecting the secure FL applications. Figure 15 (a-c) shows our observation in three domains: the subjective difference among the FL edge nodes, the location of the FL nodes, and the type of IoHT used by the FL nodes. We did not find significant effects for these three factors. In other words, the introduction of privacy and security within the deep learning FL applications only had a minor effect on these three factors. For example, the lack of proper communication bandwidth to some edge nodes affected the overall collaborative training module.

Thanks to Syed Abdullah of Advanced Media Laboratory for his great support, we identified several points of improvement. Overall, the introduction of HE added more computing cycles, which could be reduced by adding compression techniques as suggested by several researchers. Instead of sending live model gradients to the blockchain, they can be compressed in batches and then sent. The TEE enclave of professional cloud providers may suffer from GPU computation memory. Hence, we will be looking into improving the enclave computing in the coming days. We have already started the clinical trials of the secure applications with several hospitals.^2^, ^3^^2^https://www.medml.net/^3^http://advancedmedialab.com/UPM-AI-COVID19/demo-AI.html#trial

## VI. Conclusion

In this paper, we addressed the problem of adding a lightweight security and privacy algorithm that could be used within the FL ecosystem. In particular, we targeted IoHT-powered edge devices that required a privacy guarantee for privately owned health data during collaborative training as federated nodes. To prevent raw data leakage, feach federated node applied DP. Also, the amount of noise that was added for privacy protection was carefully chosen to strike a balance between the privacy budget and the accuracy degradation. HE on the edge node allowed additive and multiplicative matrix operations on deep learning operations during the FL process. The FL process was managed by a blockchain-based decentralized consensus mechanism to prevent the bias and privacy leakage of a central aggregation entity. Rather, the blockchain nodes managed the entities involved in distributed learning, the reputation and quality of each client contribution, and the storage of the intermediate and global models in a decentralized repository, i.e., the IPFS. The test results show the captured accuracy and loss metrics, which we are planning to improve in the coming days.
